# Trainee involvement in laparoscopic pyloromyotomy does not compromise outcomes: evidence from a longitudinal institutional experience

**DOI:** 10.1007/s00383-026-06435-1

**Published:** 2026-05-11

**Authors:** Sofia Martinho, Sara Nóbrega, Catarina Barroso, Jorge Correia-Pinto

**Affiliations:** 1 Pediatric Surgery Department, Hospital de Braga, Braga, Portugal; 2 Life and Health Sciences Research Institute (ICVS), School of Medicine, University of Minho, Braga, Portugal; 3 School of Medicine, University of Minho, Campus de Gualtar, 4710-057 Braga, Portugal

**Keywords:** Hypertrophic pyloric stenosis, Laparoscopic pyloromyotomy, Pediatric surgical training, Resident participation, Minimally invasive surgery, Learning curve

## Abstract

**Purpose:**

To evaluate whether supervised pediatric surgery trainees can safely perform laparoscopic pyloromyotomy (LP) and to assess the influence of trainee participation within the institutional learning curve.

**Methods:**

A retrospective single-center cohort included all infants undergoing LP between June 2011–2025. Cases were categorized as specialist- or trainee-performed (eight pediatric surgery specialists, three supervised trainees). Baseline characteristics, operative variables, surgery-related complications, incomplete pyloromyotomy, postoperative recovery, reintervention, and readmission were compared. Temporal trends and institutional CUSUM analysis were performed.

**Results:**

Seventy-seven infants were included (45 specialist-operated, 32 trainee-operated). Baseline characteristics were similar. Operative time was longer for trainees (45 versus 40 min, *p* = 0.037). Surgery-related complications occurred in 11.1% of specialist cases and in none of the trainee cases (p=0.07). All five complications (two wound infections, one incisional omental exteriorization, and two mucosal perforations) occurred with specialists. One incomplete pyloromyotomy occurred (trainees). The composite adverse outcome rate was 11.1% (specialists) versus 3.1% (trainees) (p = 0.39). Feeding progression, length of stay, reoperations and readmissions were comparable. CUSUM analysis demonstrated no adverse drift after trainee integration.

**Conclusion:**

Supervised trainees safely performed LP with outcomes comparable to specialists. Slightly longer operative time did not impact recovery. Early trainee involvement did not compromise patient safety or institutional performance.

## Introduction

Hypertrophic pyloric stenosis is one of the most frequent surgical conditions requiring intervention in early infancy [1]. Since the original description of extramucosal pyloromyotomy by Ramstedt in 1912, the procedure has become one of the most iconic and foundational operations in pediatric surgery [2]. Beyond its historical importance, pyloromyotomy holds a unique emotional and clinical resonance within the specialty: its combination of dramatic presenting symptoms, potential for life-threatening dehydration, and the almost immediate resolution that follows a successful pyloromyotomy has long made this operation uniquely rewarding for both families and surgeons, symbolizing, for many, the essence of pediatric surgical care [3].

Open pyloromyotomy performed through a right upper quadrant or circumumbilical incision has traditionally been regarded as the standard technique [1, 3]. Over the past two decades, however, laparoscopic pyloromyotomy has gained widespread acceptance and is now favored in many centers [4–7]. This shift reflects consistent evidence showing that the laparoscopic approach provides at least equivalent safety to the open technique and faster return to full feeds [8, 9]. Recent high-quality meta-analyses have confirmed comparable rates of major complications, with modest but clinically meaningful benefits favoring the laparoscopic pyloromyotomy [5, 7, 10].

As minimally invasive surgery (MIS) has evolved, the role of trainees has become an increasingly important consideration, particularly in neonatal laparoscopy, where the imperative of resident education intersects with the distinct technical challenges of small anatomy and limited operative working space. Laparoscopic pyloromyotomy is often one of the first neonatal laparoscopic operations performed by residents, yet exposure, case allocation and supervision vary substantially across training programs, contributing to heterogeneity in operative experience and competency development [11–15]. Existing evidence indicates that technical error rates may be higher when the operation is performed by clinicians without dedicated pediatric surgical training, particularly general surgery residents [16]. Conversely, young pediatric surgery trainees operating under supervision can achieve complication rates comparable to those of attending surgeons, albeit with slightly longer operative times [16, 17].

Despite these insights, the literature remains limited by small sample sizes and a lack of longitudinal evaluations of institutional learning [18–20]. Few investigations have evaluated both the early adoption of laparoscopic pyloromyotomy and the subsequent integration of trainees within a single center.

The present study assesses the impact of pediatric surgery trainee participation on the safety and perioperative outcomes of laparoscopic pyloromyotomy across more than a decade of institutional practice. By capturing both the initial phase of laparoscopic implementation and the later incorporation of trainees, this study provides an opportunity to evaluate trainee performance within the broader context of institutional learning. Given that early, structured and supervised trainee involvement—although not universally embraced in Europe or internationally—underpins our department’s educational philosophy, we aimed to determine whether this model compromises operative safety, postoperative recovery or institutional outcomes, or whether it can be implemented without jeopardizing the high standards traditionally associated with this classic operation.

## Methods

### Study design and setting

This was a retrospective observational cohort study conducted at a single tertiary pediatric surgery center. All consecutive infants undergoing laparoscopic pyloromyotomy for hypertrophic pyloric stenosis between June 2011 and June 2025 were eligible. This timeframe reflects the entire institutional experience with the laparoscopic approach since the establishment of the pediatric surgery department. No patients were excluded.

### Surgeons and grouping strategy

Procedures were performed by 11 surgeons, including 8 board-certified pediatric surgery specialists and 3 pediatric surgery trainees, under direct in-room supervision of a consultant pediatric surgeon. Operative volume varied across surgeons: specialists performed between 2 and 13 procedures each, while trainees performed between 8 and 13 procedures each. As in many pediatric surgery units, specialists in our department had heterogeneous prior exposure to laparoscopic surgery - particularly neonatal MIS - reflecting differences in training era and availability of structured MIS opportunities. As such, all had different skill levels in laparoscopic surgery. In contrast, pediatric surgery trainees in our program are exposed to minimally invasive techniques early during residency, including neonatal laparoscopy.

In our institution, structured pediatric surgical training began in January 2016 and the first laparoscopic pyloromyotomy performed by a trainee was held in February 2017, after which both specialists and supervised trainees performed pyloromyotomy. Cases were assigned to one of two groups according to the primary operating surgeon: specialist group - procedures performed by certified pediatric surgeons; trainee group - procedures in which a supervised trainee served as the primary operator.

### Patient selection and definitions

All infants diagnosed with hypertrophic pyloric stenosis based on clinical presentation and ultrasonographic criteria who underwent laparoscopic pyloromyotomy during the study period were included. No exclusion criteria were applied.

Baseline variables collected included age, sex, weight, vomiting duration, and biochemical markers of disease severity (presence of alkalosis or hypokalemia).

Operative variables included operative time, intraoperative complications, and incomplete pyloromyotomy (defined as persistent vomiting requiring corrective intervention).

Postoperative outcomes included time to full oral tolerance, length of hospital stay, reoperation, and readmission within 30 days.

Surgery-related postoperative complications were classified using a four-grade institutional scale (0 = none, 1 = minor not affecting clinical course, 2 = surgery-related requiring antibiotics or prolonging hospitalization, 3 = major complication requiring additional operative steps - e.g., mucosal perforation necessitating intraoperative repair - or reoperation). A composite adverse outcome was defined as any surgery-related complication or incomplete pyloromyotomy.

### Technique

A 5 mm laparoscopic camera is inserted via an umbilical port by the Hasson technique, with special care taken to ligate proximally the umbilical vein, in order to avoid CO2 insufflation related complications; the trocar is secured by a silk suture. A stab incision in the right upper quadrant is used to insert a 3 mm grasper, used to hold the pylorus. An arthrotomy knife is inserted via an epigastric stab incision and used to longitudinally incise the pyloric tumor from the prepyloric vein of Mayo to well up on to the antrum of the stomach. The knife is withdrawn and then a 3 mm pyloromyotomy spreader is introduced through the same epigastric incision and used to fully split the muscle. The pyloromyotomy is considered adequate when there is bulging mucosa and independent movement of the edges; the bulging mucosa is inspected for perforation. Demand feeding with the baby’s normal milk is started immediately on return to the ward and increased as tolerated.

### Temporal and learning-curve analysis

Because trainees began performing the operation only after 2017, temporal analyses were conducted to contextualize outcomes. Chronological operative times were analyzed with locally weighted regression (LOWESS), and institutional performance across time was assessed using CUSUM analysis based on the expected probability of the composite adverse outcome.

To account for institutional technical maturation, the cohort was also examined across three predefined eras: early specialist-only period (2011–2016), initial trainee integration (2017–2020) and mature institutional phase (2021–2025). This framework was used to interpret the clustering of early complications and evaluate the impact of trainee introduction.

### Statistical analysis and missing data

Statistical analyses were conducted using SPSS (version 28.0.1.0). Normality of continuous variables was assessed using the Shapiro–Wilk test and Q–Q plot inspection. Continuous variables were compared using independent-samples *t*-tests or Mann–Whitney *U* tests, depending on distribution. Categorical variables were compared using Fisher’s exact test or chi-square testing. Effect measures included mean differences with 95% confidence intervals for continuous outcomes and risk differences with 95% confidence intervals for categorical outcomes. Statistical significance was defined as two-sided *p* < 0.05. Missing data did not exceed 8% for any variable. Analyses were performed using complete available data (pairwise deletion, no imputation).

## Results

A total of 77 patients fulfilled inclusion criteria and were included in the final analysis, 45 operated on by a specialist surgeon and 32 by a resident.

Baseline demographic and preoperative characteristics were similar between groups (Table 1). Age tended to be lower in the trainee group, although this difference did not reach statistical significance. Sex distribution was identical in both groups. Weight, duration of vomiting before surgery, and the prevalence of metabolic alkalosis and hypokalemia were also comparable.

Overall complications (including non-surgical complications) occurred in 15.6% of specialist cases and 6.3% of trainee cases (*p* = 0.29), (Table 2). Surgery-related complications (grades 1–3) occurred in 11.1% of cases in the specialist group and in none of the trainee cases (*p* = 0.07). There were five surgery-related complications in total: two wound infections, one incisional omental exteriorization, and two gastric mucosal perforations. Both perforations were promptly recognized and repaired - one laparoscopically using mucosal suturing and an omental patch, and the other after conversion via an umbilical incision, with closure of the initial pyloromyotomy and performance of an adjacent one. Although this difference did not reach statistical significance, all surgery related complications occurred in procedures performed by specialists. Incomplete pyloromyotomy (one patient) occurred only in the trainee group (0% vs. 3.1%, *p* = 1.00). When both events were analyzed together, the composite adverse outcome rate was 11.1% for the specialist group vs. 3.1% for the resident group (*p* = 0.39).

Operative time was significantly longer when the primary surgeon was a trainee, corresponding to a median increase of 5 min. Time to full oral tolerance was similar between groups. Median length of hospital stay was slightly shorter in the trainee group, although this did not reach statistical significance.

Two unplanned reoperations occurred: one for incomplete pyloromyotomy (trainee group) and one for early incisional hernia with omental herniation (specialist group). Readmission occurred in one specialist-group patient (for persistent vomiting after discharge) and none in the trainee group. No mortality was recorded.

### Learning-curve and temporal institutional performance

To assess temporal trends in operative performance and the effect of trainee participation, a learning-curve analysis was performed across three predefined epochs: a specialist-only period (2011–2016, 32 patients), an early trainee-integration phase (2017–2020, 24 patients), and a mature institutional phase (2021–2025, 21 patients). Analyses were conducted at the institutional level and separately for specialists and trainees to evaluate changes in efficiency and safety over time.

**Fig. 1:**
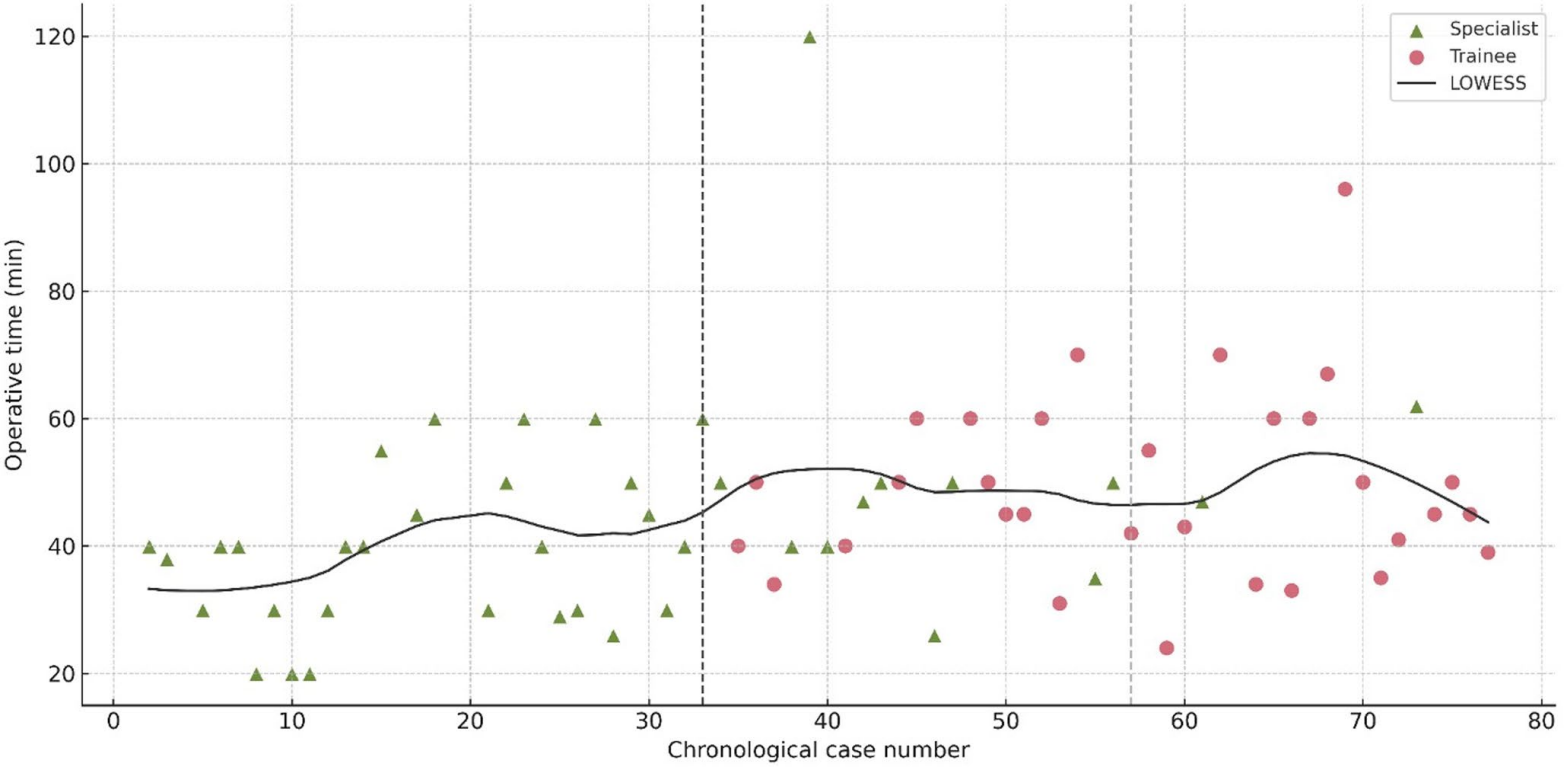
Operative time by chronological case number. Scatter plot of operative time for all laparoscopic pyloromyotomies. Green markers represent specialist-performed cases, and pink markers represent trainee-performed cases. The bold LOWESS curve illustrates overall temporal trends. The vertical dashed lines indicate 2017, when trainees first began performing the procedure under direct supervision, and 2021, marking the beginning of the third institutional period.

At the institutional level, operative times remained generally stable throughout the series, with only a modest increase after 2017, when trainees began performing the procedure (Fig. 1). Specialists maintained a stable operative-time plateau of approximately 40–50 min with low variability. Trainee operative times were understandably more variable, reflecting the supervised learning environment and the progressive inclusion, from 2017 onward, of residents in the earliest stages of their training. Importantly, however, no temporal deterioration was observed, and trainee operative times consistently remained within clinically acceptable limits throughout their participation.

**Fig. 2:**
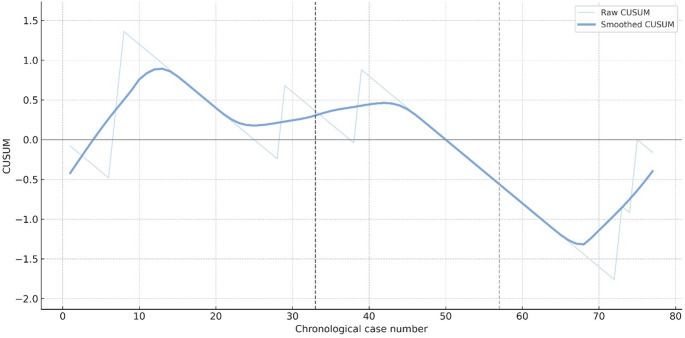
Institutional CUSUM chart (raw and smoothed). CUSUM analysis for the composite adverse outcome (surgery-related complications or incomplete pyloromyotomy), using an expected event rate of p₀ = 0.08. The light line shows raw case-by-case CUSUM values, and the bold pastel-blue curve shows the LOWESS-smoothed trend. The vertical dashed lines mark 2017 (introduction of supervised trainee participation) and 2021 (beginning of the third period). The smoothed curve remained relatively stable thereafter, indicating no sustained increase in adverse events during the training era.

Composite adverse outcomes remained consistently low across the three epochs, with rates of 9.4%, 4.2%, and 9.5%, respectively (Fig. 2). Institutional CUSUM analysis showed early fluctuations typical of initial technique adoption, followed by a prolonged plateau. Importantly, neither the raw nor the smoothed CUSUM curve displayed a sustained upward drift after trainee integration, indicating no increase in cumulative morbidity during the training era.

**Table 1:** Baseline demographic and preoperative characteristics according to surgeon type

Baseline characteristics	Total (*n* = 77)	Specialist (*n* = 45)	Trainee (*n* = 32)	*p*-value	Effect size (95% CI)
Age (days)	32.0 [22–40]	36.0 [25–42]	27.5 [19.8–38]	0.068	Median difference − 6.5 y (− 13.5 to + 0.4)
Male sex, n (%)	65 (84.4%)	38 (84.4%)	27 (84.4%)	1.000	Risk difference 0% (− 16 to + 16)
Birth weight (g)	3734.2 ± 702.4	3804.5 ± 786.8	3628.9 ± 555.4	0.268	Mean difference − 175 g (− 490 to + 138)
Vomiting duration (days)	2.0 [1–4]	2.0 [1–6]	3.0 [2–4]	0.882	Median difference + 0 d (− 1 to + 2)
Metabolic alkalosis, n (%)	30 (41.7%) (*n* = 72)	19 (47.5%) (*n* = 40)	11 (34.4%) (*n* = 32)	0.378	Risk difference + 13% (− 10 to + 34)
Hypokalemia, n (%)	8 (10.8%) (*n* = 74)	4 (9.3%) (*n* = 42)	4 (12.5%) (*n* = 32)	0.717	Risk difference − 3% (− 17 to + 12)

**Table 2:** Operative and postoperative outcomes according to surgeon type

Outcomes	Total (*n* = 77)	Specialist (*n* = 45)	Trainee (*n* = 32)	*p*-value	Effect size (95% CI)
Operative time (min)	42 [35–55] (*n* = 71)	40 [30–50] (*n* = 40)	45 [40–60] (*n* = 31)	**0.037**	Median + 5 min (+ 1 to + 14)
Intraoperative complications, n (%)	2 (2.6%)	2 (4.4%)	0 (0.0%)	0.508	Risk difference + 4% (− 3 to + 11)
Any postoperative complication, n (%)	9 (11.7%)	7 (15.6%)	2 (6.3%)	0.291	Risk difference + 9% (− 5 to + 24)
Surgery-related complications^a^, n (%)	5 (6.5%)	5 (11.1%)	0 (0.0%)	0.072	Risk difference + 11% (− 1 to + 25)
Incomplete pyloromyotomy, n (%)	1 (1.3%)	0 (0.0%)	1 (3.1%)	1.000	Risk difference − 3% (− 12 to + 6)
Composite adverse outcome^b^, n (%)	6 (7.8%)	5 (11.1%)	1 (3.1%)	0.391	Risk difference + 8% (− 4 to + 21)
Time to full oral tolerance (h)	23 [12–40] (*n* = 75)	24 [19–39] (*n* = 43)	22 [8–42] (*n* = 32)	0.143	Median + 2 h (− 6 to + 20)
Length of stay (h)	36 [24–54]	48 [24–60]	24 [24–48]	0.112	Median − 24 h (− 72 to + 2)
Reintervention, n (%)	2 (2.6%)	1 (2.2%)	1 (3.1%)	1.000	Risk difference − 1% (− 10 to + 9)
Readmission, n (%)	1 (1.3%)	1 (2.2%)	0 (0.0%)	1.000	Risk difference + 2% (− 6 to + 11)

a Surgery-related complications = postoperative grades 1–3

b Composite endpoint = surgery-related complication OR incomplete pyloromyotomy

## Discussion

This study evaluated the impact of trainee participation on outcomes of laparoscopic pyloromyotomy in a contemporary single-center cohort and found that supervised trainees performed the operation safely, with no increase in procedure-specific complications, reinterventions, or delayed recovery. Although operative time was longer in trainee-performed cases, the difference was modest and had no effect on postoperative feeding or hospital stay. These findings align with previous evidence and support the notion that, in a structured training environment, supervised trainee involvement does not compromise patient safety [16, 17, 21, 22].

Resident participation in neonatal minimally invasive surgery has been debated for more than two decades [15, 23, 24]. As laparoscopy becomes the dominant approach in many pediatric surgical units, ensuring adequate trainee exposure is essential to sustain institutional expertise and prepare surgeons for independent practice. Yet international surveys consistently highlight gaps in trainee confidence and case volume for key neonatal procedures, underscoring the need for structured, early, and supervised operative opportunities [12–15].

Evidence from other pediatric MIS domains reinforces the safety of supervised trainee participation [25]. In laparoscopic appendectomy, Baumgarten et al. demonstrated that junior pediatric surgery fellows operating under direct supervision achieved outcomes equivalent to experienced attendings [26]. A large Japanese series likewise showed that early-career surgeons without formal endoscopic certification could safely perform complex MIS procedures - including choledochal cyst excision, biliary atresia surgery, and thoracoscopic lobectomy - when directly supervised by highly experienced pediatric surgeons [27]. Complementary European data indicate that most pediatric laparoscopic procedures in high-volume centers can be safely performed by supervised trainees without increased conversion or complication rates [22]. Together, these studies highlight that operative safety in pediatric MIS depends less on surgeon seniority and more on structured supervision, deliberate skills training, and the maturity of the institutional MIS program.

Our findings for laparoscopic pyloromyotomy align with this literature. We found no significant difference in overall surgery-related postoperative complications between specialists and trainees. Incomplete pyloromyotomy occurred once in the trainee group and was promptly identified and corrected without sequelae. Conversely, all postoperative complications related to the procedure - including mucosal perforation - occurred in specialist-performed cases. Although this pattern did not reach statistical significance, it contrasts with previous reports associating complications mainly with general surgery residents lacking pediatric MIS training, but not with pediatric surgery specialists or trainees [16]. This result is unlikely to reflect case complexity: demographic and clinical baseline characteristics, including age, weight, vomiting duration, and biochemical severity, were comparable between groups, and the operation has fairly homogeneous technical difficulty.

A more plausible explanation relates to institutional learning dynamics and differences in formative MIS exposure. Specialists in our institution span a broad range of laparoscopic experience. Several completed their training before pediatric MIS was widely established and therefore approach laparoscopic pyloromyotomy at the upper limit of their comfort zone, despite strong motivation for MIS. In contrast, all trainees were embedded from the outset in a strongly MIS-oriented culture. Their training model includes early exposure to neonatal laparoscopy, progressive entrustment of procedural steps, access to ex-vivo laparoscopic simulation - shown to improve safety and accelerate skill acquisition in pediatric MIS, often outperforming traditional apprenticeship [28–30] - and systematic use of video-based learning - demonstrated to enhance technical performance and intraoperative decision-making [31]. This combination of early MIS immersion, deliberate practice, simulation, and validated video-assisted learning likely contributed to the strong performance of trainees in our cohort. These findings suggest that in neonatal MIS, operative performance is shaped less by seniority or professional status and more by the recency, continuity, and depth of MIS-focused training. In this setting, early and sustained familiarity with pediatric laparoscopy appears to be a stronger determinant of technical confidence than career stage alone.

This study has several strengths. It examines a homogeneous population undergoing a standardized minimally invasive technique. Missing data were minimal (< 8% across variables), enabling complete-case analysis. Complications were rigorously categorized, and both technical and recovery-related outcomes were evaluated. The addition of temporal and CUSUM analyses provides a richer understanding of institutional performance across different eras of the program.

Limitations include the small sample size, retrospective design, and single-center setting, which limit generalizability and statistical power. Case allocation was not randomized, and although baseline characteristics were similar between groups, unmeasured confounding cannot be excluded. The composite adverse-event endpoint, while robust, combines events with differing clinical significance.

Future research should assess whether our findings are reproducible across training environments with differing structures, expectations, and supervisory models [32]. Our institution reflects a southern European paradigm in which residents train exclusively in pediatric surgery from the outset, without general surgery residency or graded fellowship stages, and where early exposure to neonatal MIS is increasingly incorporated. Comparative analyses with systems such as the United States (with a staged training pathway- where hierarchical fellow–resident supervision models are frequently encountered and operative autonomy is often granted later in training) [33], or with the United Kingdom, France, and Germany (which use mixed or step-wise pediatric surgical training models) [30, 34, 35], may help clarify how differing educational philosophies shape institutional learning curves and patient safety. Multicenter, cross-system studies would be valuable to identify training frameworks that best balance safety with the acquisition of independent MIS competency.

Taken together, our findings support the view that the philosophy of early, structured, and well-supervised trainee involvement adopted in our department does not compromise individual patient safety or institutional outcomes in laparoscopic pyloromyotomy. Instead, it produces surgeons who are progressively more confident and proficient in neonatal MIS as this approach continues to evolve as the standard of care. In a procedure that is technically delicate yet profoundly rewarding - given its dramatic symptom resolution and immediate impact on both infants, families and surgeons - this model maintains motivation and enthusiasm for future generations of pediatric surgeons. Ultimately, trainees in pediatric MIS should be regarded as developing peers and collaborative partners whose structured participation strengthens both patient care and the long-term vitality of the specialty.

## Data Availability

No datasets were generated or analysed during the current study.
